# Psychiatry in UG curriculum of medicine: Need of the hour

**DOI:** 10.4103/0019-5545.37312

**Published:** 2007

**Authors:** M. Thirunavukarasu

**Affiliations:** Department of Psychiatry, Stanley Medical College and Hospital, Chennai - 600 001, Tamil Nadu, India

Planning for health is a relatively new activity in most developing countries. Planning for mental health is an even more recent activity. In 1975, WHO released its first report on mental-health service planning in developing countries. If you really follow it up, little action has been taken; and if any action has been taken, it has been very patchy.

Certain heads of state believe that mental illness is not at all present in their country and it is a problem of the West. Not all politicians and health administrators subscribe to these views, but until recently many of them had rather narrow and negative views about mental-health care. In most people's minds mental illnesses are equated with madness, and the popular belief is that such patients should be locked up in hospitals. Few realize and acknowledge most common mental-health problems are dependence on tobacco and alcohol, depression, anxiety, childhood problems, sexual dysfunction, sleeping and eating disorders rather than psychoses, which are the most conspicuous illnesses.

Take the rate of suicide and attempted suicide and its prevalence; this clearly indicates high prevalence of underlying depression and its inadequate treatment. Lakhs of people, mostly young, have died because of suicide. Furthermore, a large number of persons have been displaced and exposed to political, religious and other conflict situations over the past few decades. Take the disaster like tsunami, which necessitated hunting for mental-health workers or service personnel.

Psychiatric epidemiology in India - Moving beyond numbers is the topic of a chapter in the recently released book “Mental health - An Indian perspective”. India with its population of more that one billion faces myriad health problems. The increase in per capita income, along with changing healthcare availability, with control of nutritional and infectious disorders, has resulted in greater life expectancy of people. The scenario has also brought to the fore the new challenge of behavior-linked manmade lifestyle-related problems. In this evolving health-care scenario, noncommunicable diseases pose major problems due to lack of skilled health-care manpower, inadequate information and inability of the system to meet this challenge all over the world, including India. The aggressive, market-oriented liberalizing economy in combination with an invasive media has helped in rapid expansion of hazardous life styles. The social, biological and psychological strengths of the past have been slowly replaced by a fragile life pattern of people, making them more vulnerable to social, mental and psychological problems at all ages.

Mental-health problems have long been recognized in every society. Communities had their own mechanisms of handling these problems, many of which are gradually being replaced by modern science. A greater understanding of mind and behavior in all dimensions has revolutionized our efforts of managing problems in today's society. The interaction of man's mind and behavior is at an exciting phase today, with advances in genetic, molecular, biochemical and environmental domains based on agent-host environment approach. The organization of service for those with mental-health problems has moved from crude primitive methods to more sophisticated technological approaches with a combination of pharmacological and nonpharmacological methods.

The Indian medical education system is one of the largest in the world and consists of 262 medical schools, each associated with a university producing 28,000 doctors each year. One-third of these doctors leave India every year for residency and/or practice abroad, with around 1,500 medical graduates immigrating to the USA each year to enter residency training. The quality of Indian medical education and the physicians it produces therefore has implications for the USA and the entire world.

A number of high-profile issues confront undergraduate education in India, a country with a long history of medicine. They include curriculum reform, including the structure of internship; proliferation of new medical schools; accreditation of new medical schools; selection of medical students and faculty development. The important issue confronting medical education in India is curriculum reform, with many calls for change in curriculum having been made in the past 30 years. In the mid-1970s, the Shrivastav Committee, a group of educators commissioned by the Indian government, advocated reorientation of medical education in accordance with national needs and priorities. They recommended a medical education committee to implement reforms. In 1986, the Bajaj committee; and in 1993, Katker and Adkoli advocated updated course content, revisions in student assessment and innovative teaching methodology. To implement these changes, they suggested faculty development, establishing medical education units, making educational funding more transparent. These recommendations were reiterated in 2004 by Majumdar in a government-commissioned report, in which he emphasized the need for political commitment and leadership to achieve relevant, evidence-based medical education.

The government, as well as the ministers, is well aware about the prevalence and burden of mental-health issues in our country. It all began with fire; given the context of the tragedy of Erwadi, the knee-jerk reaction of administrative and judicial inquisition made the honorable Supreme Court give direction to the government to improve the mental-health care facilities in the country. The national mental-health program increased allocation of funds to improve the infrastructure facilities to provide mental-health care, thanks to the dynamic Union Health Minister Thiru. Dr. Ambumani Ramadoss.

The Indian Psychiatric Society (IPS) on its own has around 4,000 psychiatrists as its members. Majority of the psychiatrists are based in urban areas and belong to the private sector. The IPS, as well as the Indian Association of Private Psychiatry, readily came forward to promote mental health and alleviate the sufferings of the disadvantaged mentally ill persons. This will not suffice and cannot meet the ends. Mental illness is a chronic illness that has to be followed up and rehabilitated. So the psychiatrists cannot cater to the needs of our people. Mental-health care must be available at the primary-care level; only then can we cater to the burden of mental illnesses and mental-health problems. Thus the number of mental-health professionals is clearly inadequate for the people of our country.

It is therefore better and advisable to teach Psychiatry at the undergraduate level itself so that all physicians in the country will have some knowledge about mind, mental health and mental-health problems and so on. Majority of cardiovascular, diabetic and gastrointestinal problems are managed well by the primary-care physicians. In the same way, mental-health problems must be managed by primary-care physicians. For this, Psychiatry must be included in the curriculum of undergraduate training.

Any significant reforms to the Bachelor of Medicine and Bachelor of Surgery (MBBS) curricula must ultimately be approved by the Medical Council of India (MCI), a governmental agency established in 1934 under the Ministry of Health and Family Welfare. The MCI stipulates in significant detail the rules of medical college curriculum, its structure and content. In addition MCI regulates allocation of time among disciplines, percentage of lecture time, required percentage of class attendance, etc. The university conducts the examination as per the MCI norms, and the assessments are controlled by the parent university.

Hence the Indian Psychiatric Society should address and apprise the members of the Medical Council, as well as the authorities of the parent university, about the need and necessity to include psychiatry in the UG curriculum of medicine in collaboration with Government of India and the Ministry of Health and Family Welfare. Only then can the proposal be put forth in the general council and executive council of MCI and approved by the Government of India and the Ministry of Health and Family Welfare. Following this, the syllabus and other modalities of the subject will be worked out and implemented. Hence all the members of the IPS should take this up as a personal agenda - to apprise the members of the Medical Council from their respective areas and states so that when the matter comes for discussion in the Council, it can find its place of importance and significance. The task force is working in this direction.
